# Occupational burnout among obstetrics and gynaecology residents: a systematic review

**DOI:** 10.3389/fpubh.2025.1666659

**Published:** 2025-10-23

**Authors:** Sylwia Szcześniewska, Kornelia Zaręba, Wojciech Stefan Zgliczyński, Michał Ciebiera, Stepan Feduniw

**Affiliations:** ^1^Second Department of Obstetrics and Gynecology, Center of Postgraduate Medical Education, Warsaw, Poland; ^2^Warsaw Institute of Women’s Health, Warsaw, Poland; ^3^Obstetrics and Gynecology Department, College of Medicine and Health Sciences, United Arab Emirates University, Al Ain, United Arab Emirates; ^4^School of Public Health, Center of Postgraduate Medical Education, Warsaw, Poland; ^5^Obstetrics and Gynecology Department, Spital Uster, Uster, Switzerland

**Keywords:** burnout, residency, gynaecology, obstetric, anxiety, depression, stress

## Abstract

**Introduction:**

Occupational burnout, a complex condition frequently affecting highly committed individuals, is characterized by persistent emotional, mental, and physical exhaustion. The demanding nature of specialization in obstetrics and gynaecology makes residents particularly vulnerable to burnout. This study aimed to evaluate the mental and emotional well-being of residents during their specialization in obstetrics and gynaecology, as well as their susceptibility to stressors and the resulting burnout.

**Materials and methods:**

A systematic literature review was conducted in accordance with PRISMA guidelines. The PubMed, Web of Science, and Cochrane Library databases were searched to identify peer-reviewed articles examining the mental and emotional well-being of physicians during their residency in obstetrics and gynaecology their exposure to stressors and associated risk of burnout. Studies published before 30 August 2024 were included in the review.

**Results:**

A total of 16 studies were analysed, revealing that the prevalence of burnout among obstetrics and gynaecology residents ranges from 46 to 86%. The studies indicate that residency in obstetrics and gynaecology significantly increases the risk of burnout, driven by factors such as psychological stress, young age, long working hours, female gender, and lack of support.

**Conclusion:**

The persistently high incidence of burnout among obstetrics and gynaecology residents underscores the urgent need for targeted preventive measures and enhanced mental health support within this field. Immediate action is required to address these issues by challenging stigma, reforming residency structures, improving both workplace and personal environments, and implementing comprehensive strategies to support residents’ mental well-being, with particular attention to emotional exhaustion, depression, and suicidal ideation.

**Systematic review registration:**

https://www.crd.york.ac.uk/PROSPERO/view/CRD420251067594, identifier CRD420251067594.

## 1 Introduction

Medical training requires continuous self-improvement and dedication under challenging circumstances, often involving critical decisions that impact human lives. It is also a long and demanding career path (1). Prolonged working hours, night shifts, and frequent exposure to life-threatening patient situations contribute significantly to the psychological and emotional burden placed on physician (2). While many physicians are able to manage this kind of stress effectively, its cumulative impact inevitably affects their personal lives and overall well-being, sometimes leading to burnout (3, 4).

Burnout is defined by a triad of emotional exhaustion (EE), depersonalization (DP), and reduced personal accomplishment (PA) (5). Individuals experiencing burnout typically exhibit diminished enthusiasm for their work, increased apathy toward social interactions, and a pervasive disengagement from both personal and professional responsibilities. The manifestation of these symptoms often coincides with dissatisfaction with job duties, frustration, and negative attitudes toward patients, colleagues, and one’s personal life (6, 7). Burnout primarily affects motivated and committed individuals who are deeply engaged in their professional duties. Owing to the consistently high-stress nature of clinical practice—characterized by continuous responsibility, time constraints, and substantial workloads—physicians are particularly vulnerable to burnout (8).

Nearly 40 to 75% of physicians may experience burnout at some point in their careers, suggesting that virtually all physicians are at risk of encountering this syndrome during their professional lives (3, 4). Training in obstetrics and gynaecology is associated with a higher risk of burnout compared to other specialties (9, 10). This specialty combines surgical complexity with critical prenatal care, encompassing responsibilities that range from intricate surgical procedures to holistic, emotionally sensitive support for pregnant individuals during the antepartum, intrapartum, and postpartum periods (10). Expectations regarding maternal care are exceptionally high, with public and patient scrutiny—often emotionally charged—intensifying the stress and moral pressure placed on clinicians.

The study is conducted in order to assess the risk of physician burnout across all medical disciplines, particularly in obstetrics and gynaecology and among physicians in training or residency. It is hoped that the burden experienced by young residents will be alleviated by the findings of this study, and support will be provided during the demanding period of their training. Both healthcare systems and individual healthcare organisations should invest greater time and effort in implementing evidence-based measures, what we hope to underline within this systematic review.

The objective of the study was to evaluate existing research on the mental and emotional well-being of residents during their specialization in obstetrics and gynaecology, as well as their vulnerability to burnout risk factors.

## 2 Materials and methods

### 2.1 Study design

This systematic review was conducted in accordance with the Preferred Reporting Items for Systematic Reviews and Meta-Analyses (PRISMA) guidelines (11). The PRISMA checklist is included as Supplementary Table 3. The protocol was registered in the PROSPERO database under the registration number CRD420251067594.

### 2.2 Research question

The study’s Research Question was “How does occupational burnout affect physicians during their specialisation in gynaecology and obstetrics?”

### 2.3 Search strategy

Two independent reviewers (S.S. and S.F.) independently screened potentially eligible publications. Any disagreements were resolved through discussion or, if necessary, adjudicated by a third reviewer (M.C.). The literature search was conducted in PubMed, Web of Science, Embase, and the Cochrane Library up to 30 August 2024. The screening of grey literature, as Google Scholar yielded approximately 4,000,000 results, making it unfeasible to review systematically. Therefore, it has been decided not to include grey literature in our analysis.

The Mendeley Reference Manager was used for data analysis.

The following keywords were used: “burnout” AND (“doctor” OR “resident” OR “physician”). We also screened the databases using the following MeSH terms: “Burnout, Professional[MeSH] AND Internship and Residency[MeSH]AND Physicians[MeSH] AND Obstetrics[MeSH] AND Gynecology[MeSH].” Only studies assessing the impact of burnout on gynaecologists were evaluated and included in the review. The reference lists of the selected studies were thoroughly examined to identify additional potentially relevant articles. To avoid redundancy, only the most recent or comprehensive reports from the same authors were included. Additionally, the reference lists of relevant papers and systematic reviews were reviewed to identify further potentially eligible studies.

### 2.4 Inclusion and exclusion criteria

Eligible studies were required to meet all of the following inclusion criteria:

1. Investigation of mental well-being, stress levels, and symptoms of occupational burnout.
2. Physicians specializing in obstetrics and gynaecology as the study population.
3. Original research including a comparison group from other medical specialties.

Studies that met any of the following exclusion criteria were omitted:

1. Research that did not report any of the specified outcomes.
2. Studies lacking a control group.
3. Studies not published in English.
4. Articles in formats such as editorials, newspaper pieces, or other forms of popular media

### 2.5 Data extraction and quality assessment

**Figure 1:**
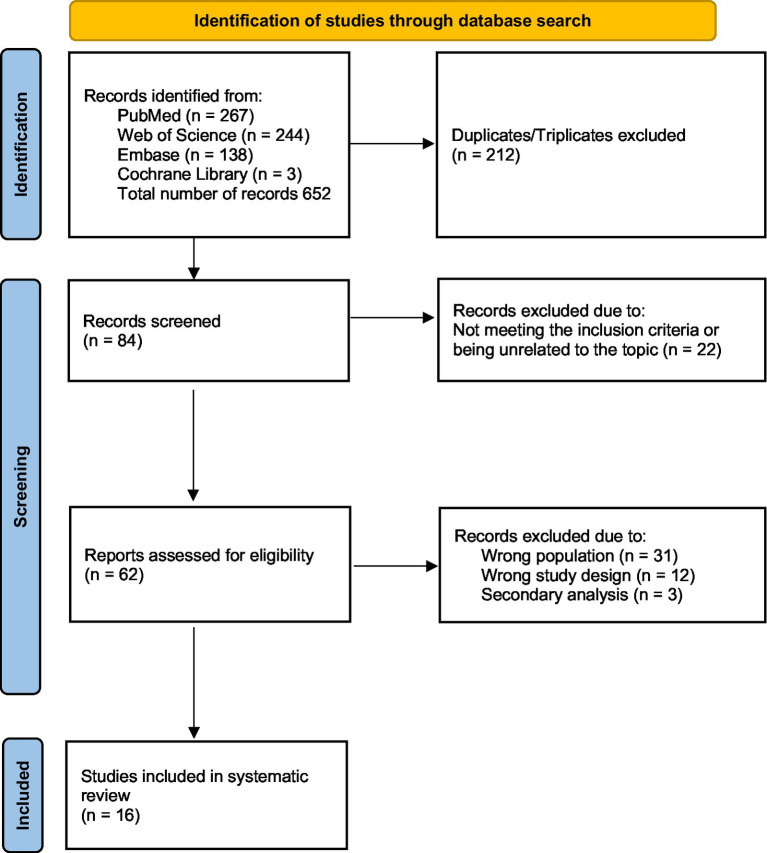
PRISMA flow diagram.

Two independent reviewers (S.S. and S.F.) performed data extraction using a predetermined data extraction form developed by S.F. All discrepancies or disagreements were resolved through discussion with a third reviewer (M.C.). The information extracted from the eligible studies included the following: study characteristics (first author, publication year, country of origin, study design, and research groups) and data concerning the study populations. The methodological quality of the included studies was assessed using the Newcastle–Ottawa Quality Scale (12). Studies with a score of 7 points or higher were considered high quality. The risk of bias was evaluated, on the study level, independently by two writers (S.F. and S.S.), with a third reviewer (M.C.) adjudicating any disparities in the selection procedure. The majority of the studies considered were of moderate to high quality and presented in Table 1. To deliver a thorough picture of burnout among gynaecology and obstetrics residents, we incorporated research of inferior quality and elevated bias risk, which are also mentioned in Table 1. A quantitative synthesis could not be conducted due to the heterogeneity of the included research. A comprehensive comparative summary of the findings is presented. A comprehensive summary of the search and screening process is presented in Figure 1.

**Table 1:** Included studies not reporting prevalence and risk factors of burnout.

Study	Country	Burnout scale	Number of participants		Outcomes	Additional findings
			Study group	Control group		
Nazeema et al. (22)	South Africa	Maslach Burnout Inventory Human Services Survey (MBI-HSS) and PHQ-9	19 residents in obstetrics and gynaecology	318 residents in other specializations (surgery, internal medicine, pathology, anaesthetics, paediatrics, radiology, nuclear medicine, psychiatry, emergency medicine, public health)	46% of all included residents had burnout. Risk factors of burnout and depression: - being a resident (RR 2.2, 95%CI: 1.5–3.2) - disciplines of anaesthetics (RR 1.4, 95%CI: 1.03–1.9) and obstetrics and gynaecology (RR 1.5, 95%CI: 1.1–2.1) - low experience (RR 2.2, 95%CI: 1.51–3.22) - prior psychiatric diagnosis (RR 1.47, 95%CI: 1.15–1.38)	The occurrence of depression correlates with burnout. Many physicians self-prescribe medicines to manage burnout and depression symptoms.
Kashif et al. (23)	Pakistan	Oldenburg Burnout Inventory (OLBI)	36 residents and 19 specialists in obstetrics and gynaecology	87 members of medical personnel in various specialized areas (surgeons, nurses, surgery technicians)	39% of healthcare providers in obstetrics and gynaecology exhibited significantly elevated levels of burnout during the COVID-19 pandemic. Risk factors of burnout: - advanced age - longer working hours	Exhaustion was a more accurate marker for burnout than disengagement
Elhadi et al. (21)	Sudan	Maslach Burnout Inventory Human Services Survey (MBI-HSS)	26 residents in obstetrics and gynaecology	182 residents in other specializations (internal medicine, paediatrics, urology, surgery, ENT, oncology, dermatology, and psychiatry)	86% of all residents had symptoms of burnout. Risk factors of burnout: - extended working hours [MBI-HSS score mean 3.13 ± 0.94 (*p* < 0.001)] - residency in pediatrics residents [MBI-HSS score mean 3.19 ± 0.86 (*p* < 0.001)] and obstetrics and gynaecology [MBI-HSS score mean 3.08 ± 0.86 (*p* < 0.001)]	Neither year of residency nor marital status influenced burnout occurrence.
Nimer et al. (19)	Jordan	The Copenhagen Burnout Inventory (CBI)	26 residents in obstetrics and gynaecology	455 residents in other specializations (internal medicine, general surgery, pediatrics, forensic medicine, psychiatry, dermatology, and pathology)	78% of all residents had burnout. Risk factors for burnout: - psychological stress (*β* 2.34, 95%CI: 1.88–2.81) - residency in obstetrics and gynaecology (*β* = 9.66, CI: 3.59–15.73) - longer working hours - 51–75 h / week (*β* 4.07, 95%CI: 0.52–7.62) - 76–100 h / week (*β* 7.27, 95%CI: 2.86–11.69) - > 100 h / week (*β* 7.27, 95%CI: 0.06–14.49)	Providing mental health insurance to residents, increasing wellness and job safety awareness, expanding vacation and sick leave benefits, implementing night float systems, hiring more residents for specialties with heavier workloads, and encouraging a safety culture where residents understand their rights at work may reduce the burnout occurrence.
Cheng et al. (14)	USA	Maslach Burnout Inventory	14 residents in obstetrics and gynaecology	91 residents in other specializations (family medicine, internal, pediatric, surgery)	79% of obstetrics and gynaecology residents experienced burnout. Risk factors of burnout: - personal mistreatment (OR 7.6, 95%CI: 1.7–34.4)	Residents who experienced personal mistreatment were also more prone to express symptoms of anxiety and depression (OR 3.8, 95%CI: 1.6–9.1).
Ye et al. (18)	China	Maslach Burnout Inventory	338 obstetricians and gynaecologists	338 paediatrics	57% of obstetricians and pediatricians had signs of occupational burnout. Risk factors of burnout - comunication problems between patients and doctors - increased number of working hours - low family support	
Bourne et al. (20)	UK	Maslach Burnout Inventory	1,364 residents in obstetrics and gynaecology	1738 specialists	43% of residents had burnout symptoms Risk factors of burnout: - young age - MD from the UK or Ireland (aOR 1.74, 95%CI: 1.41-2.16).	Protected factors of burnout is another ethnicity than Caucasians. Burnout is closely linked to anxiety (OR 3.59, 95%CI: 3.07-4.21), depression (OR 4.05, 95%CI: 3.26-5.04), suicidal thoughts (OR 6.37, 95%CI: 3.95-10.7), anger/irritability (OR 3.51, 95%CI: 3.0-4.1), sleep problems or insomnia (OR 3.15, 95%CI: 2.70-3.67) and substance abuse (OR 2.57, 95%CI: 1.71-3.89).
Ironside et al. (26)	USA	None	10 residents in obstetrics and gynaecology	62 residents in other specializations (family medicine, internal medicine, pediatrics, and psychiatry).	No percentage of burnout was described. Risk factors of burnout: - a sense of meaning at work - fatigue and exhaustion - cultural norms in medicine - the steep learning curve from medical school to residency - social relationships in the workplace and personal life	Although interventions at the individual level are significant, there is an urgent necessity for systemic reforms addressing workload, autonomy, recognition, community, and the integration of personal and professional ideals.
Gyorffy et al. (25)	Hungary	Maslach Burnout Inventory	166 doctors in obstetrics and gynaecology	4,140 doctors in all specializations.	No percentage of burnout was described. Risk factors for burnout: - being single - long working hours - shift work - increased number of workplaces	65% of doctors exhibited a moderate to mild level of personal success, whereas 49% showed a moderate to severe level of emotional weariness, and 46% experienced depersonalization.
Chen et al. (24)	Taiwan	Maslach Burnout Inventory	90 doctors in obstetrics and gynaecology	719 residents in other specializations (general medicine, general surgery, paediatrics, emergency medicine, neurological surgery, dermatology, anaesthesiology, ophthalmology, orthopaedics)	No percentage of burnout was described. Risk factors of burnout: - being a resident - long working hours (13-17 h continuously/day or ≥41 h/week) - history of depression - hospital culture of work - not appropriate patient-physician relations - being married - younger age (<30 years)	Higher levels of burnout were linked to more frequent drinking brought on by an overwhelming workload. There was a considerable correlation between the degree of emotional tiredness and the number of medical errors. Between 2008 and 2012, 7 physician deaths due to karoshi (death from overwork) were reported.
Prins et al. (17)	Netherlands	Maslach Burnout Inventory	11 residents in obstetrics and gynaecology	147 residents in other specializations (anaesthesiology, cardiology, dermatology, laryngology, genetics, internal medicine, medical microbiology, neurology, ophthalmology, pathology, paediatrics, psychiatry, radiology, radiotherapy, rehabilitation medicine, and surgery)	13% of medical residents met the criteria for burnout. The highest incidence of burnout is observed among psychiatric residents.	Gynaecology residents reported markedly higher levels of personal success compared to their counterparts, particularly psychiatrists.
Martini et al. (15)	United States	Maslach Burnout Inventory	36 residents in obstetrics and gynaecology	285 residents in other specializations (internal medicine, neurology, ophthalmology, dermatology, general surgery, psychiatry, family medicine)	50% of all residents fulfilled the criterion for burnout. Obstetrics and gynaecology is the highest at 75%, while family medicine is the lowest at 27% risk of burnout. Factors associated with burnout: - first year of residency - being single - personal stress - dissatisfaction with faculty	In order to reduce burnout and promote healthier academic and clinical environments. Training reforms that target the first year of residency with regular monitoring of burnout could be the most effective.

## 3 Results

**Figure 2:**
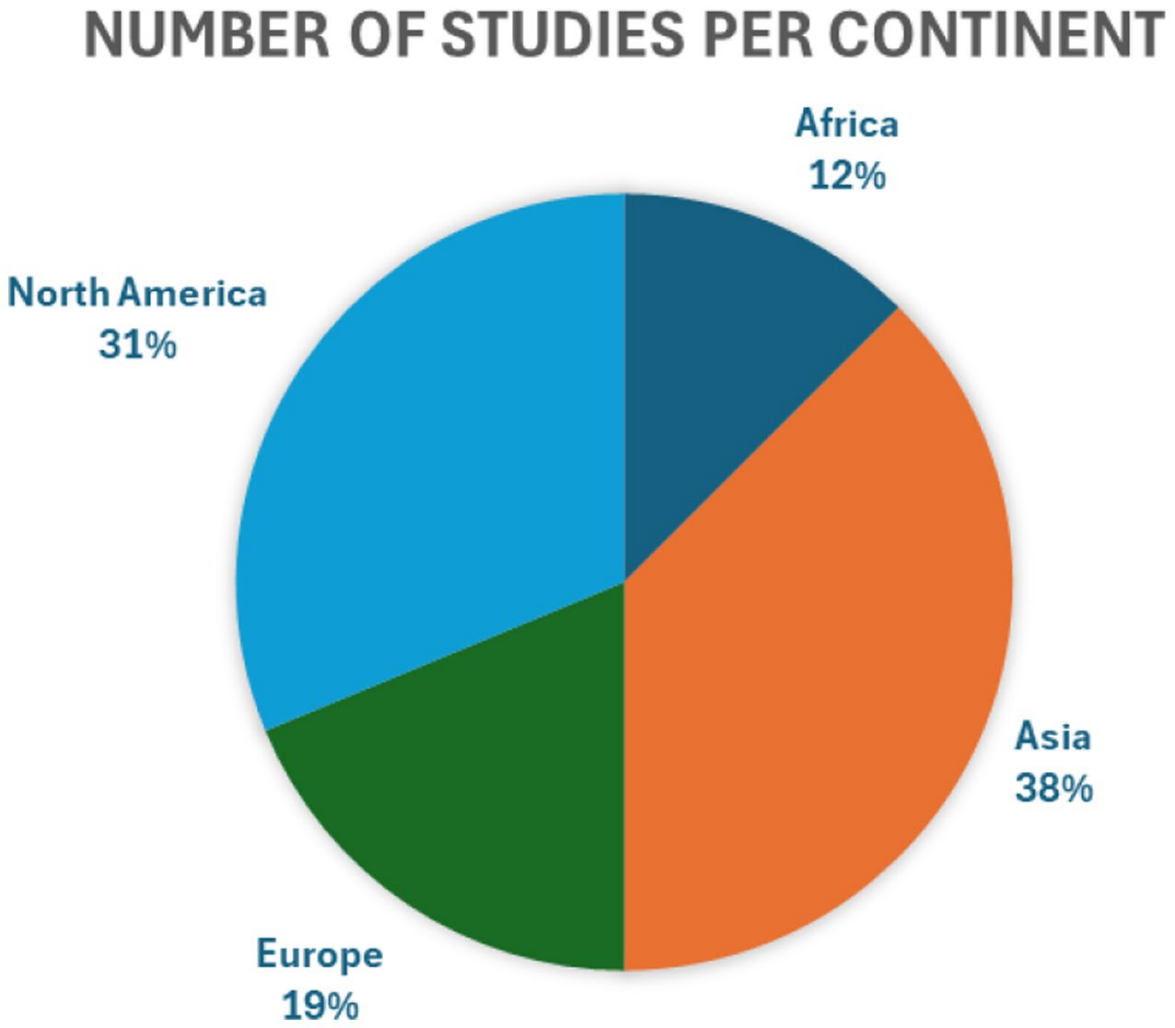
Number of studies per continent.

The review included 16 studies involving physicians in obstetrics and gynaecology (13–27). Ten of these studies provided a comparison between residency in gynaecology and other specialties (14–17, 19, 21, 22, 26–28). In contrast, six studies did not clearly specify whether the comparison involved residency in gynaecology versus other residencies, or a general comparison between gynaecologists and physicians from other specialties (13, 18, 20, 23–25). The studies demonstrated overall high quality, encompassing 2,267 gynaecologists and 8,891 physicians from other specialties (13–28). The studies analysed come from 4 continents, as shown in Figure 2. A detailed description of the included studies is provided in Table 2. Studies that did not assess risk factors were also included and are presented in Table 1 and Figure 2.

**Table 2:** Included studies reporting prevalence and risk factors of burnout.

Study	Country	Burnout scale	Number of participants		Outcomes
Scheid et al. (13)	USA	Professional Fulfillment Index (PFI)	50 doctors in obstetrics and gynaecology	43 doctors in neonatology	Physician burnout and other indicators of psychological well-being may be enhanced following a yoga and mindfulness intervention.
Fernando et al. (28)	Sri-Lanka	The Copenhagen Burnout Inventory (CBI)	18 residents in obstetrics and gynaecology	227 residents in other specializations (internal medicine, surgery, paediatrics, emergency medicine, orthopaedics, psychiatry, anaesthesia)	The determinants linked to a diminished probability of personal burnout included a high frequency of healthy habits (OR 0.2, 95%CI: 0.1–0.5), satisfaction with skill development opportunities from training programs (OR 0.4, 95%CI: 0.2–0.9), and regular utilization of deep learning approaches during study (OR 0.2, 95%CI: 0.1–0.5).
Agha et al. (16)	Saudi Arabia	MBI-HSS	29 residents in obstetrics and gynaecology	67 residents in other specializations (internal medicine, emergency medicine, general surgery, nephrology, paediatrics)	89% of respondents exhibited significant emotional exhaustion at 69%, high depersonalisation at 64%, and poor personal accomplishment at 39%.
Ghetti et al. (27)	USA	MBI, Psychological Medicine Inventory, Jefferson Scale of Physician Empathy	15 residents in obstetrics and gynaecology	2 specialists in obstetrics and gynaecology	70% of residents had moderate to severe burnout both at baseline and 12 months following the initiation of Balint training

Residents in obstetrics and gynaecology, compared with those in other medical specialties, exhibit higher levels of burnout and specific psychological vulnerabilities. Nazeema et al. reported an increased risk of burnout (RR 1.5, 95% CI: 1.1–2.1) (20), as did Nimer et al. (*β* = 9.66, CI: 3.59–15.73) (17). Similar associations were also identified by other authors (14–17, 19, 21, 22, 26–28). Burnout rates among obstetrics and gynaecology residents ranged from 46% to over 85%, with emotional exhaustion and depersonalization being particularly prominent (14, 15, 19, 21, 22). For example, Elhadi et al. (21) reported burnout in 86% of obstetrics and gynaecology residents in Sudan, while Agha et al. (16) found emotional exhaustion in 69% and depersonalization in 64% of a similar cohort in Saudi Arabia.

Most studies described obstetrics and gynaecology as one of the disciplines most susceptible to burnout (13–16, 18–27). Longer working hours were identified as a significant risk factor for physician burnout (19, 21, 23–25). This was typically defined as working more than 10 consecutive hours per day or over 40 h per week (14). Additional risk factors reported by Martini et al. (15), Cheng et al. (14), and Kashif et al. (23) included younger age, female gender, single marital status, lack of institutional support, and exposure to mistreatment.

Chen et al. (24) and Ye et al. (18) indicated that working conditions such as prolonged shifts, unsupportive hospital environments, and poor patient–physician relationships play critical roles in the development of burnout. Despite higher levels of personal accomplishment, the overall trend points to significant emotional strain (17). Furthermore, large-scale analyses conducted by Bourne et al. (20) and Gyorffy et al. (25) highlighted the association between burnout and suicidal ideation, depression, and increased intent to migrate among younger gynaecologists.

## 4 Discussion

According to the present review, a significant proportion of physicians specializing in obstetrics and gynaecology experience burnout during their residency. Longer working hours (over 40 h per week) and prolonged shifts were identified as a significant risk factor for physician burnout. Additional risks included younger age, female gender, single marital status, lack of institutional support, unsupportive hospital environments, poor patient–physician relationships, and exposure to mistreatment. Moreover, we found an association between burnout and suicidal ideation, depression, and increased intent to migrate among younger gynaecologists.

The emergency nature of the specialty, irregular and extended working hours, including night shifts and on-call duties, are among the most predisposing factors for occupational burnout among obstetrics and gynaecology residents. A similar association has been observed among residents in anaesthesiology and emergency medicine (4, 22, 29). Prolonged working hours have been identified as a significant risk factor for burnout among physicians (19, 21, 23–25). This is typically defined as working more than 10 consecutive hours per day or over 40 h per week (14). A 2001 survey by Defoe et al. (30) reported that obstetrics and gynaecology trainees worked between 61 and 100 h per week. More recent findings by Nimer et al. (19) revealed that, despite improvements in working conditions, over 30% of residents still reported working more than 75 h per week. Moradi et al. (31) identified an elevated risk of occupational burnout among obstetrics and gynaecology residents, identifying analogous risk factors, including quantitative work overload, role conflicts connected to work, and inadequacies in employment resources. Nonetheless, the reliability of this comparison must be regarded with caution, given that the overall quality of the research incorporated in their systematic analysis was rather low. Our evaluation primarily focused on recently published articles. It included studies utilising various tools for assessing burnout levels, such as the Oldenburg Burnout Inventory (OLBI) and the Maslach Burnout Inventory (MBI).

Extended working hours often stem from systemic issues such as inadequate staffing, inefficient workflows, and excessive administrative burdens. These factors can exacerbate clinicians’ feelings of being unsupported amid overwhelming demands, thereby intensifying workplace stress. According to studies by Nimer et al. (19), Martini et al. (15), Ironside et al. (26), and Gyorffy et al. (25), experiencing personal stress is significantly associated with an increased risk of burnout.

Burnout symptoms are most commonly observed in younger physicians with limited clinical experience, particularly those in the early stages of their training (14, 15, 22, 23). According to Bourne et al. (20), greater professional experience, older age, and progression within a specialty have been identified as protective factors. Over time, colleagues often develop more effective coping strategies like physical activity or emotional and physical distancing from work, enabling them to manage stress more successfully and reduce their vulnerability to burnout (32).

The organizational culture in many healthcare institutions may foster competitive dynamics, hierarchical barriers, and poor communication—factors that undermine mutual trust and collegial support. Cheng et al. (14) and Martini et al. (15) further report that dissatisfaction with the work environment, as well as experiences of mistreatment in the workplace, are key psychosocial risk factors for burnout. The specific demands of the field, particularly extended work hours and night shifts, combined with insufficient psychosocial support, strained interpersonal relationships, and systemic inefficiencies, significantly contribute to stress, emotional exhaustion, depersonalization, and reduced professional efficacy (4, 33). These core components of burnout contribute to frustration and decreased professional efficacy.

Physician burnout is associated with compromised patient care across various stages of healthcare delivery. Our findings indicate that physicians experiencing burnout are more likely to be involved in patient safety incidents, deliver substandard care due to diminished professionalism, and receive lower patient satisfaction ratings. Depersonalization appears to have the most detrimental impact on the quality and safety of patient care, as well as on patient satisfaction. Physicians exhibiting these symptoms are more prone to diminished patience, empathy, and energy, which undermines the quality of patient care and weakens the patient–physician relationship. Research by Chen et al. (24), Ironside et al. (26), and Ye et al. (18) has identified strained interpersonal relationships—both between physicians and patients, and among workplace colleagues—as significant risk factors for burnout and as consequences of its progression. Relational difficulties within the clinical environment may not only contribute to the onset of burnout but also exacerbate its symptoms, thereby creating a self-perpetuating cycle of professional and psychological decline.

In addition to impaired psychosocial relationships with both patients and colleagues, young physicians face other serious challenges associated with burnout, including suicidal ideation, depression, and an increased propensity to emigrate among younger gynaecologists (20, 25). Moreover, physicians experiencing burnout frequently report a diminished sense of professional identity, reduced confidence in their problem-solving abilities, and feelings of isolation and misunderstanding. Research by Nazeema et al. (22) and Chen et al. (24) highlights that a personal or family history of psychiatric disorders even more predisposes individuals to burnout during residency. A psychiatric history, whether personal or familial, can increase susceptibility to the recurrence of mental health disorders and the emergence of new psychiatric symptoms during periods of prolonged psychological stress and overwhelming circumstances (34). The majority of the studies analysed identified obstetrics and gynaecology as one of the disciplines most susceptible to burnout (13–16, 18–27). However, Prins et al. (17) found that obstetricians and gynaecologists reported significantly higher levels of personal accomplishment compared to psychiatrists. Despite relatively high levels of personal accomplishment, symptoms of occupational and personal burnout, particularly in the context of inadequate psychosocial relationships, are associated with considerable emotional strain (17).

Psychosocial factors play a critical role in the development of occupational burnout. Poor psychosocial conditions are partly a consequence of limited time for recovery and social engagement, often resulting from prolonged work shifts that may leave inadequate time for family life, personal interests, or professional development. According to studies by Nimer et al. (19), Martini et al. (15), Ironside et al. (26), and Gyorffy et al. (25), being single is a risk factor for burnout among present groups. As previously noted, the absence of a fulfilling personal life and limited opportunities for recovery outside of work are closely associated with reduced professional satisfaction, which can contribute to the onset of burnout.

Considering the geographical distribution of the studies presented, four were conducted in the United States (13–15, 26, 27). The private social insurance system in the United States may constitute an additional cause of occupational burnout (35). The necessity to adapt appropriate diagnostic and therapeutic methods to the patient’s financial means may constitute a strong stressor. The clash between the idealistic assumptions held during academic training and the realities of the healthcare system functioning may contribute to significant psychological distortions among young physicians. Another contributing factor is the high rate of litigation observed in the United States, which contributes to increased anxiety, diminished job satisfaction, and defensive behaviour among physicians (36). Numerous studies have also been conducted in Muslim-majority countries (Pakistan, Sudan, Jordan, Saudi Arabia), where medical law is grounded in strict Muslim legislation, which may involve the risk of imprisonment in the event of a patient’s death (16, 19, 21, 23). In line with this theory, the highest rate of burnout, exceeding 80%, was observed in traditional Islamic contexts such as Sudan and Saudi Arabia (16, 21). On the other hand, the lowest prevalence of occupational burnout was observed among residents in the Netherlands (13%), where a quite liberal blame-and-shame system for medical error and a national health insurance system prevails- findings which may corroborate the previously presented theory (17).

### 4.1 Possible solutions and coping strategies

Burnout not only undermines the quality and safety of patient care but also negatively impacts physicians’ mental health, increasing the risk of depression, suicidal ideation, and intentions to emigrate. While individual-level initiatives—such as healthy lifestyle habits, Balint groups, and mindfulness training—offer protective benefits, they must be complemented by structural changes addressing workplace culture, workload distribution, and professional recognition. A range of coping strategies and preventive measures has been shown to mitigate the risk of burnout among medical trainees. Fernando et al. demonstrated that a high frequency of healthy lifestyle habits—such as regular physical activity and balanced nutrition—is significantly associated with a lower risk of burnout, as is satisfaction with skill development opportunities provided by training programs (28). The consistent use of deep learning techniques during study has also emerged as a protective factor. According to Scheid et al. (13), mindfulness-based interventions—including yoga and structured programs such as the Respiratory One Method—have been effective in enhancing resilience and emotional regulation. Ghetti et al. (27) also report that Balint group training has been shown to provide lasting benefits, thereby enhancing emotional well-being. Rodrigues et al. suggest that health interventions—such as duty-hour reductions, mindfulness training, psychiatry-guided self-development groups, and mantra-based meditation—are effective in reducing burnout among residents in high-demand specialties, including general surgery, anaesthesiology, obstetrics and gynaecology, and orthopaedics (37). Meanwhile, social and organisational factors remain the most influential contributors to differences in burnout prevalence across medical specialties, beyond individual-focused interventions, underscoring the need for systemic modifications in clinical training environments (38). Ironside et al. (26) emphasised the importance of structural reforms targeting workload, autonomy, recognition, sense of community, and alignment between personal and professional values.

Addressing burnout requires a comprehensive approach that includes cultural changes promoting empathy, collaboration, and sustainable working conditions, alongside mental health support, organisational reform, and equitable workload distribution. Despite often being overlooked in mental health research, the significant clinical and emotional demands faced by gynaecology residents increase their vulnerability to stress and burnout. Legislation should prioritise funding and regulations aimed at supporting physician well-being, particularly in high-risk specialties such as obstetrics and gynaecology. Future research should focus on longitudinal studies to assess the effectiveness of specific interventions and to identify causal pathways. As residency training is highly resource-intensive, greater attention should be given to preventing burnout among residents in obstetrics and gynaecology.

A significant limitation of the included studies was their cross-sectional design, which limited the ability to establish causal relationships between stress and burnout outcomes. Moreover, many studies failed to clearly distinguish between burnout in specialists and residents, or did not specify the exact composition of comparison groups, potentially undermining the accuracy of subgroup analyses. The comparison groups varied across studies, as obstetrics and gynaecology residents were compared with different specialties. Additionally, variability in findings may have resulted from inconsistencies in the definitions of burnout and the measurement tools used.

## 5 Conclusion

This research highlights burnout as a critical issue during medical training, with increased prevalence among obstetrics and gynaecology residents. The findings underscore the urgent need for coordinated legislative initiatives and both systematic and institutional solutions to prioritise the well-being of physicians, particularly those in obstetrics and gynaecology training. A range of strategies must be explored to protect physicians’ mental health and reduce the risk of burnout. The well-being and sustainability of the medical workforce—as well as the quality and safety of patient care—depend on a comprehensive approach to addressing burnout.

## Data Availability

The original contributions presented in the study are included in the article/Supplementary material, further inquiries can be directed to the corresponding author.
